# A seroepidemiological survey of Crimean Congo hemorrhagic fever among Cattle in North Kordufan State, Sudan

**DOI:** 10.1186/1743-422X-10-178

**Published:** 2013-06-05

**Authors:** Ibrahim A Adam, Mubarak AM Mahmoud, Imadeldin E Aradaib

**Affiliations:** 1 Department of Clinical Medicine, Molecular Biology Laboratory (MBL), Faculty of Veterinary Medicine, University of Khartoum, P.O. Box 32, Khartoum North, Sudan

**Keywords:** Epidemiology, Survey, Viral hemorrhagic fevers, CCHFV, ELISA, Sudan

## Abstract

**Background:**

Crimean Congo hemorrhagic fever (CCHF), caused by CCHF virus (CCFV), may cause a fatal hemorrhagic illness in humans with mortality rate of approximately 30%. However, in animals the disease is typically asymptomatic and no clinical hemorrhagic infections appears to be associated with CCHFV. Recently, CCHF activity has been detected in western and southern Kordufan region, Sudan. Currently, no information is available in regard to previous exposure of livestock to CCHFV infection in the region.

**Aims:**

In the present study, a seroepidemiological survey was conducted to determine the prevalence of CCHF and to identify the potential risk factors associated with the disease among cattle in North Kordufan State, Sudan.

**Methods:**

In this survey, 299 blood samples were collected randomly from six localities in North Kordufan State and were tested by enzyme-linked immunosorbent assay (ELISA) for detection of CCHFV-specific immunoglobulin G (IgG) antibodies.

**Results:**

The result of the study indicated that the prevalence rate of CCHF was relatively high among cattle, where serological evidence of the infection was observed in 21 (7.0%) of 299 animals. Older cattle were eight times more likely to be infected with the virus (OR=8.0824, CI=1.174-66.317, p-value=0.034). Cross breeds were at 37 time higher at risk compared to endogenous breed (OR=37.06, CI=1.455-944, p-value=0.029). Highly tick-infested cattle are 6 times higher at risk for CCHF when compared to tick-free animals (OR=6.532, CI=1.042-10.852, p-value=0.030).

**Conclusion:**

It is recommended that surveillance of CCHF should be extended to include other ruminant animals and to study the distribution of ticks in the region to better predict and respond to CCHF outbreak in the State of North Kordufan, Sudan.

## Introduction

Crimean-Congo hemorrhagic fever (CCHF) is a tick-borne disease caused by CCHF virus (CCHFV) of the genus *Nairovirus* in the family *Bunyaviridae*. The CCHFV infection is transmitted to humans by tick bites, handling of ticks, exposure to blood or tissues of viremic livestock, or direct contact with blood and bodily fluids of infected patients. Ticks of the genus *Hyalomma* are the primary vectors for CCHFV, and the virus is endemic throughout Africa, the Middle East, Eastern Europe, and central Asia [1-10]. Recently, CCHFV has been repeatedly reported as an important emerging infectious viral pathogen in the Kordufan region, Sudan. We reported the first outbreak of CCHF in 2008 among health care workers in Alfulah rural hospital, West Kordufan [11]. Subsequently, another outbreak was reported in 2009 in Donkup village, Abyei District, South Kordufan [12]. Very recently, a nosocomially acquired CCHFV infection was reported in an attending physician in North Kordufan as a result of providing medical care to CCHFV infected patient from Lagawa, an area of endemicity in South Kordufan. However, CCHF has never been recognized in North Kordufan State [13]. Earlier serologic studies have suggested the presence of various arboviruses in Sudan, including CCHFV [14,15]. Indirect serologic evidence of CCHFV infection was recorded in camels exported from Sudan to Egypt [16] and in sheep and goats exported to Saudi Arabia [17]. It is well documented that viremia and CCHFV-specific antibodies develop in infected livestock including, sheep, cattle and camels. However, the infection is typically asymptomatic and no clinical hemorrhagic disease appears to be associated with CCHFV in infected livestock [18]. Never the less, infected livestock, particularly cattle could provide virus for tick-borne transmission to highly susceptible humans; thus, play an important role in the epidemiology of the disease [18-20]. It is, therefore, becoming increasingly obvious that the control of emerging viral pathogens, such as CCHFV, is especially important in the Sudan given the large numbers of livestock in the country, and their importance to the national economy and rural communities. Therefore, epidemiologic studies including implementation of improved surveillance are urgently needed to better predict and respond to this devastating disease in the Kordufan region, Sudan [21]. The objectives of the present study were to estimate the prevalence of CCHFV infection and to identify the potential risk factors associated with the disease among cattle in North Kordufan State, Sudan.

## Results

The result of this survey showed that out of 299 animals, 21 were found to be infected with CCHF indicating that the overall prevalence rate was 7% among cattle in North Kordufan State. The highest and the lowest rate of infection were recorded in Umrawaba (10.3%) and Abuzabad (3.5%), respectively. The individual risk factors attributes indicated that older cattle were eight times more likely to be infected with CCHFV (OR=8.0824, CI=1.174-66.317, p-value=0.034). Cross breeds are highly susceptible to tick infestation and they were at 37 time higher at risk compared to endogenous breed (OR=37.06, CI=1.455-944, p-value=0.029). The management risk factors attributes showed that highly tick-infested cattle are 6 times higher at risk for CCHF when compared to tick-free animals (OR=6.532, CI=1.042-10.852, p-value=0.030). The results are summarized in (Table 1). In contrast, there was no

**Table 1:** **Logistic regression analysis showing significant difference (p**<**0**.**05) between CCHFV seropositive cattle and risk factors (age**, **breed and number of tick per animal) associated with the disease in North Kordofan State**, **Sudan**

**Risk factors**	**OR**	**95.0% C.I**	**p-value**
Age	8.82	1.17 - 66.32	0.034
Breed	37.06	1.46 - 94.43	0.29
Ticks number	6.53	1.04 - 10.85	0.030

 significant difference between CCHFV seropositive cattle and other individual or management risk factors included in the study such as, animal sex, body condition, animal source, grazing system, other animals in the herd, herd size, farm yard, vector control, tick treatment, tick control, milk production, history of diseases and localities. The results are shown in (Table 2).

## Discussion

Previous studies on experimental CCHFV inoculation of cattle showed that infected animals amplified the virus to a sufficient level to infect the tick vector. The infected cattle developed a low-titre viremia and became seroconverted [18,22,23]. Thus, during the viremic stage, cattle can provide virus for tick-borne transmission to highly susceptible humans [1,24,25]. This finding suggested that cattle may play an important role in the epidemiology of the disease. In contrast, CCHFV causes highly fatal infection in humans with 30% mortality. Among different ruminant species present in Sudan, cattle were selected for this study as CCHFV infection appears to occur most frequently in larger mammals, which are the preferred hosts of adult tick vector, Hylomma species [26]. The occurrence of nosocomial CCHF outbreaks worldwide and the risks these cases pose for medical staff in resource poor health care facilities, necessitate the importance of improved surveillance system for this important emerging viral pathogen [6,11,12,27-30].

In the present investigation, the risk factors that were significant in the univariable model were re-entered in logistic regression whenever a new risk factor becames significant. In the final models, a variable with a P-value <0.05 was considered statistically significant. The final

**Table 2:** **Logistic regression analysis showing lack of association (p**-**value** >**0**.**05) between CCHFV seropositive cattle and other risk factors in North Kordofan state**, **Sudan**

**Risk factors**	**OR**	**95**.**0**% **C**.**I**	**p**-**value**
Animal sex	3.03	0.66-13.45	0.15
Grazing system	1.02	0.27-38.34	0.99
Body condition	1.11	0.17-7.22	0.94
Animal source	0.39	0.03-5.69	0.49
Other animals	0.65	0.14-2.93	0.57
Herd size	0.76	0.01-1.37	0.99
Farm yard	2.31	0.71-7.57	0.16
Tick problem	3.5	0.03-6.27	0.94
Vector control	1.92	0.13-3.61	0.72
Tick treatment	0.45	0.05-4.49	0.50
Milk production	1.28	0.42-3.97	0.66
Disease history	0.78	0.09-2.37	0.87
Localities	0.10	0.01-4-44	0.24

 models of CCHFV seropositive cattle included only three independent risk factors were statistically significant. There was significant difference between the CCHFV infection rate and the age of the animal. When assessing age as risk factor, it was shown that the calves started to get infected with CCHF after the age of 2 years. At this age, the animals are usually released into the pasture for grazing, where they are likely to be exposed to infected tick and subsequent CCHFV infection. We believe that the association of CCHFV infection rate and age is probably attributed to frequent exposure of older cattle to infected tick in the pasture. In contrast, young calves are usually kept indoors and are well taken care of by the animal owners from infectious diseases particularly, tick-borne infections. Previous epidemiological surveys indicated that there were higher risks of older cattle for CCHFV infections in different countries including Egypt, Iran and Turkey [20,23,31]. In addition, there was also a significant difference between the breed of the animal and CCHFV infection rate. The highest infection rate was observed among cross breeds (high percentage of exotic blood) as they are highly susceptible to tick infestation and subsequent CCHFV infection. In contrast, the indigenous breeds are relatively resistant to tick infestation hence, they were at lower risk for the disease compared to cross breed. Moreover, there was a significant difference between the number of ticks per animal and the rate of the infection with the disease. It is well documented that CCHF is a tick-borne zoonotic disease and thus, heavily tick-infested cattle are likely to become CCHFV positive by bites of infected ticks [23,25]. Many species of mammals can transmit CCHFV to ticks when they are viremic [24,25]. It should also be noted that treatment of cattle with insecticides should be applied monthly to prevent tick infestation. The lack of association between seroprevalence and tick treatment is probably due to the infrequent treatments with insecticides by the nomads specially, under field conditions. This phrase has been inserted into the text of the discussion section. Small vertebrates such as hares and hedgehogs, which are infested by immature ticks, may be particularly important as amplifying hosts [1]. Whereas some avian species such as ostrich can amplify the virus others are refractory to infection but may act as mechanical vectors by transporting infected ticks over a long distance. Migratory birds might spread the virus between distant geographic areas [32,33]. In contrast, the risk assessment studies indicated that there was no significant difference between CCHFV infection and the rest of the individual or management risk factors included in the study. It is worth mentioning that the gender has no significant difference for CCHFV infection among male and females as both sexes are equally infected with the disease. Likewise, there was no significant difference between localities and CCHFV infection rate, suggesting wide distribution of the tick vector all over the localities of North Kordufan.

## Conclusions

The scientific data presented in this study indicated that CCHFV does exist in North Kordufan State and that humans are at risk of becoming infected with the disease. Never the less, an outbreak of CCHF among humans in this region of the Sudan is yet to be reported. It is recommended that livestock attendants and slaughter house workers should consider strict hygienic measures when handling tick-infested livestock or their associated products. The residents have to be aware of the risk of being infected with CCHFV as a result of consumption of uncooked animal-derived products. This is because the social habit of consumption of raw offals of cattle and raw livers of sheep with bile salts and spices (mararah) is not uncommon in different regions of the Sudan, which may serve as a means of transmission of CCHFV [34]. Physicians and medical health workers in North Kordufan should consider this virus in their efforts to diagnose the disease in patients with clinical presentations compatible with those of CCHFV. Future surveillance program for CCHF should be extended to include other susceptible animals such as sheep, goats and camels. In addition, the high-risk human groups (animal attendants, slaughter house workers and butchers), and the distribution of ticks in the region should also be considered to better predict and respond to CCHF outbreak in the State of North Kordufan, Sudan.

## Methods

### Study area

**Figure 1:**
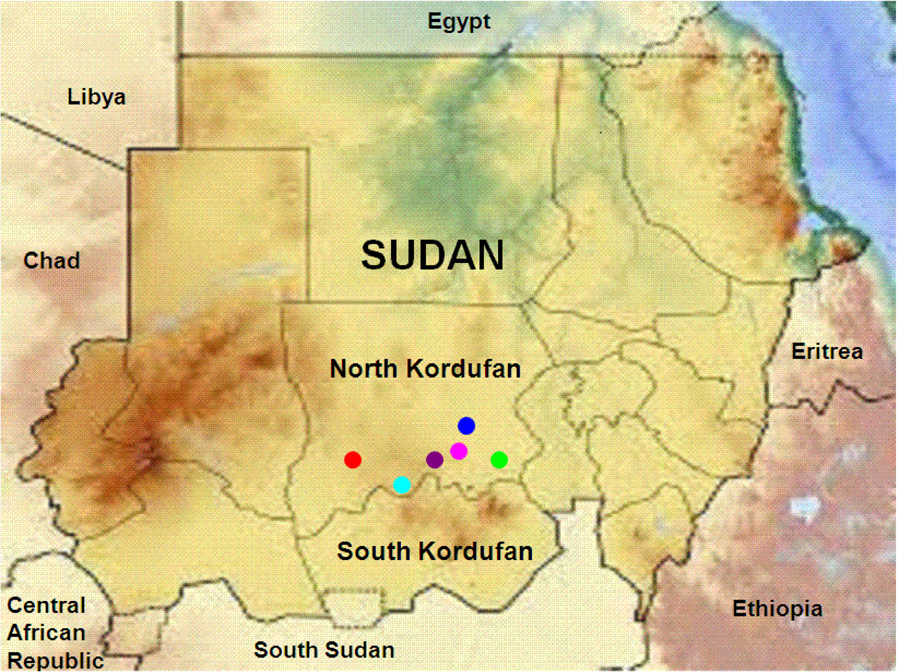
**Map of Sudan showing six localities in North Kordufan State included in the study.** “Green circle symbol”= Umrawaba; “Pink circle symbol” = Sheikan; “Blue circle symbol”= Barah; “Violet circle symbol”= Elkhwei; “Light-Blue cirle symbol”= Abuzabad; “Red circle symbol”= Ennuhud.

The North Kordofan State is located between longitudes 27.00 and 32.20 East and latitudes 12.12° and 16.4° North, occupying an area of 190,840 square km. The State is boarded by North Darfur State in the north-west; Northern State in the north; Khartoum State in the east; White Nile State in the south-east; West Kordofan state in the south-west and South Kordofan State in the south. The total population in North Kordufan is approximately 02.9 million. About 63%, 13% and 24% are rural, urban and nomadic people, respectively. The livestock population in North Kordufan constitutes one of the major sources of the income to the national economy and rural communities. Recent official livestock population was estimated to be 960, 500 for cattle; 7,200,000 for sheep; 3,600,000 for goats; and 1,200,000 for camels. The map of Sudan showing the localities in North Kordufan is shown in (Figure 1).

### Study design

A cross sectional study was conducted to estimate the prevalence rate of CCHF in cattle and to investigate the

 potential risk factors associated with the disease. The multistage probability sampling method was conducted. Six localities in North Kordufan were randomly selected from all nine localities in North kordufan (Figure 1). Two administration units were selected from each locality. Seven villages were selected from each unit. Finally, simple random sampling was applied to choose the animals from each herd [21].

### Questionnaire

A pre-tested structured questionnaire with the primary objective of elucidating the multifactorial background of disease was conducted in an interactive manner at all selected herds. All animals included in this study were subjected to a questionnaire, which was filled out by the animal owners. The questionnaire included individual risk factors attributes age (younger animals < 2years, older animals 2 years and above), sex (male, female), breed (indogenous, exotic), previous history of the disease (previous exposure to CCHF, no exposure), body condition (Thin, fats), and management risk factors attributes herd size (small, medium and large), grazing system (nomadic, seminomadicand stationary), milk production (high, low and none), vector control (use of insecticide or not), Tick treatment frequency (less than 1month, 1–3 months and, more than 3 months). The source of each animal in the herd (Raised on farm, Purchased from other farms or Purchased from local market).presence of other animal species in the herd, (the present of other animals such as sheep, goat and camels in the cattle herd). Herd size of cattle (small, medium and large).

### Ethical clearance

The study received ethical clearance from the Research Board of the Faculty of Veterinary Medicine, Sudan University for Science and technology, Khartoum, Sudan. The risk factor information was obtained from the animal owners through the questionnaire form, which permitted use of the samples for diagnostic and research purposes.

### Collection of blood samples

A total of 299 serum samples of cattle were collected randomly from six localities in North Kordofan state, Sudan. These localities include (Umrawaba, Barah, Sheikan, Ennuhud, Elkhuwei and Abuzabad). Blood samples were collected from the jugular veins in clean sterile vaccutainers and were allowed to clot and sera were separated and kept frozen at -20°C until used.

### Enzyme-linked immunosorbent assay (ELISA)

Indirect enzyme-linked immunosorbent assay (ELISA) was performed to screen the sera for CCHFV-specific immunoglobulin G (IgG) antibodies basically as described by [22]. ELISA was performed in 96-well immunoassay microplates (Nunc, Roskilde, Denmark) and optimal working dilutions of reagents were determined by chessboard titration. Unless stated otherwise, 100 uL test volumes were used, incubations were performed for 1 h at 37°C. The plates were washed three times with PBS containing 0 I% Tween 20 (Merck, Darmstadt, Germany) (PBST), wells were post-coated with 200, ul of PBS containing 2% bovine serum albumin (Calbiochem, La Jolla, USA), and the diluent for reagents was PBS containing 10% Skimmed milk (Amba, Denmark). Briefly, the plates were coated with sucrose-acetone extracted CCHFV antigen and incubated overnight at 4°C. The source of the antigen used is cell lysate from CCHFV Nigeria strain IbAr 10200. The details for the preparation of the CCHFV antigen were basically as described by Mariner et al. [35]. The antigen used in this study was obtained from the Center for Disease Control and Prevention, Atlanta, USA). The plates were washed, and aliquots of test sera (positive and negative controls) were added in separate wells at a dilution of 1:100. After a 1-h incubation, the plates were washed, and rabbit anti-bovine IgG conjugated with horse radish peroxidase (HRP) was added to the plate at a dilution of 1,000 and incubated for 1h. The plates were then washed and the substrate, 2,2′-azino-bis(3-ethylbenthiazoline-6-sulfonic acid, (Kirkegaard and Perry Laboratories) was added. CCHFV-infected cattle serum sample was incorporated in each ELISA plate as positive control to estimate the higher limit of the sensitivity. Negative control sera were obtained from CCHFV-free animals and from cattle infected with Rift valley fever virus (RVFV), a related viral hemorrhagic fever virus to estimate the lower level of the specificity of the ELISA assay. The results were read either visually or by using ELISA reader set at 405 nm. A presumptive diagnosis was made when IgG antibody in the test sample had a significant color change or had higher optical density than the ratio between the positive and negative controls.

### Statistical analyses

The data were entered in computer using statistical package for social sciences (SPSS) software package for window (version 16.0) and double checked before analyses. Logistic regression analyses were performed using the seropostive cattle to CCHFV Ig G as dependent variable and the risk factors as independent variables. Odd ratios and 95% confidence interval were calculated and P value <0.05 was considered significant.

## Competing interests

The authors declare that they have no competing interests.

## Authors’ contributions

IAA collected the blood samples and optimized the ELISA for detection of IgG antibodies in cattle sera, and prepared the draft manuscript. MAM collected blood samples designed the study. IEA designed the experiment and prepared the final manuscript. All authors read and approved the final version of the manuscript.
